# New Therapeutic Approaches for Alzheimer’s Disease and Cerebral Amyloid Angiopathy

**DOI:** 10.3389/fnagi.2014.00290

**Published:** 2014-10-20

**Authors:** Satoshi Saito, Masafumi Ihara

**Affiliations:** ^1^Department of Regenerative Medicine and Tissue Engineering, National Cerebral and Cardiovascular Center, Suita, Japan; ^2^Department of Neurology, National Cerebral and Cardiovascular Center, Suita, Japan

**Keywords:** Alzheimer’s disease, cerebral amyloid angiopathy, treatment, perivascular drainage, cilostazol

## Abstract

Accumulating evidence has shown a strong relationship between Alzheimer’s disease (AD), cerebral amyloid angiopathy (CAA), and cerebrovascular disease. Cognitive impairment in AD patients can result from cortical microinfarcts associated with CAA, as well as the synaptic and neuronal disturbances caused by cerebral accumulations of β-amyloid (Aβ) and tau proteins. The pathophysiology of AD may lead to a toxic chain of events consisting of Aβ overproduction, impaired Aβ clearance, and brain ischemia. Insufficient removal of Aβ leads to development of CAA and plays a crucial role in sporadic AD cases, implicating promotion of Aβ clearance as an important therapeutic strategy. Aβ is mainly eliminated by three mechanisms: (1) enzymatic/glial degradation, (2) transcytotic delivery, and (3) perivascular drainage (3-“d” mechanisms). Enzymatic degradation may be facilitated by activation of Aβ-degrading enzymes such as neprilysin, angiotensin-converting enzyme, and insulin-degrading enzyme. Transcytotic delivery can be promoted by inhibition of the receptor for advanced glycation end products (RAGE), which mediates transcytotic influx of circulating Aβ into brain. Successful use of the RAGE inhibitor TTP488 in Phase II testing has led to a Phase III clinical trial for AD patients. The perivascular drainage system seems to be driven by motive force generated by cerebral arterial pulsations, suggesting that vasoactive drugs can facilitate Aβ clearance. One of the drugs promoting this system is cilostazol, a selective inhibitor of type 3 phosphodiesterase. The clearance of fluorescent soluble Aβ tracers was significantly enhanced in cilostazol-treated CAA model mice. Given that the balance between Aβ synthesis and clearance determines brain Aβ accumulation, and that Aβ is cleared by several pathways stated above, multi-drugs combination therapy could provide a mainstream cure for sporadic AD.

## Introduction

Alzheimer’s disease (AD) is the most common cause of dementia in the elderly. AD is pathologically characterized by β-amyloid (Aβ) plaques within the brain parenchyma and Aβ accumulation in blood vessels (cerebral amyloid angiopathy; CAA), as well as by the formation of neurofibrillary tangles and neurodegeneration (Duyckaerts et al., 2009). AD was not previously thought to be closely linked to cerebrovascular disease (CVD), but accumulating lines of evidence have shown a strong relationship between AD and vascular dementia (VaD) (Fotuhi et al., 2009; Kalaria and Ihara, 2013). AD and CVD share common risk factors (Viswanathan et al., 2009; Kalaria et al., 2012), and treatment of vascular risk factors is associated with slower decline in cognitive impairments of AD patients (Deschaintre et al., 2009). The Nun study revealed that CVD plays an important role in determining the presence and severity of the clinical symptoms of AD (Snowdon et al., 1997). Aβ accumulation and other AD changes are also recognized in elderly patients without apparent dementia (Funato et al., 1998; Schneider et al., 2007), which implies a strong relationship between AD neuropathology and the aging processes. Many reports have described that a majority of sporadic dementia patients have a mixture of AD and CVD pathology (Neuropathology Group of Medical Research Council Cognitive Function and Aging Study (MRC CFAS), 2001; Toledo et al., 2013). Hemorrhage, infarctions, and vascular changes are not specific indicators for VaD.

Cerebral amyloid angiopathy often induces lobar hemorrhage and cortical microhemorrhage, which mainly affects the occipital lobe (Charidimou et al., 2012). In addition, imaging technology advances, including 7 T MRI, have identified numerous cortical microinfarcts (CMI), which have been attributed to CAA (Suter et al., 2002; van Veluw et al., 2013; Westover et al., 2013). Cognitive impairment in AD patients may result from hypoperfusion/ischemia and CMIs, as well as synaptic disturbance and neuronal loss caused by Aβ and tau accumulation (Okamoto et al., 2009; Launer et al., 2011; Smith et al., 2012). Small vessel injury is frequent in both AD and VaD. CAA was previously thought to be pathologically different from Binswanger disease, one of the common forms of VaD characterized by arteriolosclerosis and white matter change. However, Binswanger disease and CAA are now often regarded as part of the same spectrum disease; the former labeled type 1 and the latter type 2 small vessel disease (Pantoni, 2010). Both types of arteriopathies make dementia patients vulnerable to hemodynamic fluctuation through impairments in cerebral autoregulation and vascular reactivity (Tanoi et al., 2000; Pimentel-Coelho and Rivest, 2012). Consequently, hypoperfusion induces Aβ overproduction and elimination failure (Zlokovic, 2011; Carare et al., 2013; Elali et al., 2013). Brain ischemia and hypoxia modulates amyloid precursor protein (APP) cleavage enzymes such as β-secretase and γ-secretase, thereby resulting in increased Aβ production (Sun et al., 2006; Guglielmotto et al., 2009; Kitaguchi et al., 2009; Li et al., 2009). Excess Aβ contributes to the impairment of Aβ clearance and CAA (Joachim et al., 1989; Rovelet-Lecrux et al., 2006; Han et al., 2008). Aβ elimination failure could also result from arteriolosclerosis (Weller et al., 2009). Thus, dementia patients with a single simple etiology are scarcely seen, except for juvenile familial AD cases caused by mutations in the APP or presenilin genes, comprising <1% of AD cases (Campion et al., 1999).

**Figure 1:**
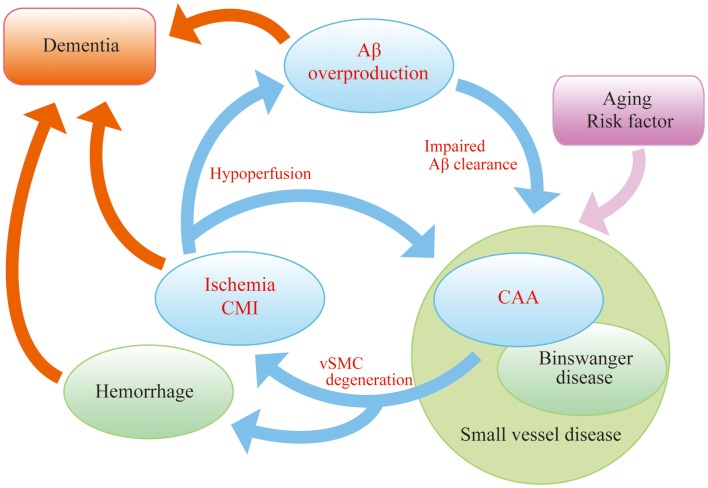
**“AD malignant cycle”**. Aβ overproduction impairs Aβ elimination leading to vascular smooth muscle cells (vSMC) degeneration, cerebral ischemia, and microinfarcts. Ischemia also induces Aβ overproduction. Such vicious circle consists of core pathology in sporadic AD. Note that cessation of Aβ overproduction is not sufficient to sever the cycle.

In order to explore novel therapies in AD, we must consider the “AD malignant cycle” (Figure 1). In this scheme, cessation of Aβ overproduction is not sufficient to treat patients with sporadic AD, and important components of the cycle, brain ischemia, and CAA should also be noted. Insufficient Aβ clearance seems to be more crucial than Aβ overproduction in sporadic AD patients (Mawuenyega et al., 2010). Even in familial AD cases, the onset of dementia is often delayed until the fifth or sixth decade, suggesting that the aging-associated failure in clearance also plays a part in the pathogenesis of inherited types of the disease (Weller et al., 2008). Therefore, recent work has focused on the failure of Aβ elimination as the most important therapeutic targets and adopted a “neurovascular” approach as a strategy to tackle AD (Vardy et al., 2005; Deane et al., 2008; Carare et al., 2013).

This review mainly focuses on the mechanisms of Aβ elimination and the drug development to facilitate Aβ clearance. The perivascular lymphatic drainage system, one of the Aβ clearance mechanisms, is closely associated with AD and CAA (Carare et al., 2013). In addition, the possibility of drugs enhancing perivascular drainage as well as future strategies for AD and CAA treatment will be reviewed.

## Aβ Clearance: 3-d Mechanism

**Figure 2:**
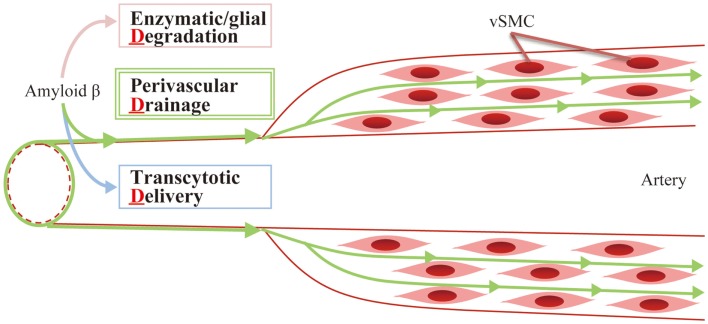
**Aβ clearance: 3-d mechanism**. Aβ is mainly eliminated by the following mechanisms: (1) enzymatic/glial degradation, (2) transcytotic delivery, and (3) perivascular drainage.

So far, several mechanisms of eliminating Aβ proteins have been identified, which fall into three main categories (3-“d,” Figure 2):

- Enzymatic/glial degradation
- Transcytotic delivery
- Perivascular drainage

## Enzymatic/Glial Degradation

Aβ catabolism is regulated by a series of degrading enzymes as well as glial cells, such as astrocytes and microglia, in the brain parenchyma (Vardy et al., 2005). Among them, neprilysin has received much attention (Iwata et al., 2000). Previous reports have described impaired Aβ degradation in neprilysin-deficient mice (Iwata et al., 2001) and amelioration of Aβ pathology in APP-transgenic mice, when injected with viral vector expressing human neprilysin gene (Marr et al., 2003; Iwata et al., 2004, 2013). Levels of neprilysin mRNA were found to be significantly lower in the hippocampus and middle temporal gyrus of AD brains compared with normal control patients (Yasojima et al., 2001). Decreased neprilysin activity was also associated with CAA (Miners et al., 2006). Thus, the up-regulation of cerebral neprilysin activity could potentially be targeted in the treatment of AD. Indeed, a somatostatin receptor agonist has recently been shown to increase neprilysin activity and decrease Aβ levels in senescence-accelerated mice (Sandoval et al., 2012). However, Meilandt et al. reported that an 11-fold greater neprilysin overexpression failed to reduce pathogenic Aβ oligomers and improve deficits in spatial learning and memory in AD model mice (Meilandt et al., 2009). It was also reported that cerebral Aβ concentration was too low to be degraded by neprilysin (Shibata et al., 2000). The affinity of neprilysin for its physiological substrates (e.g., enkephalins, tachykinins, atrial natriuretic peptide) is within the millimolar range (Hersh and Morihara, 1986), while the levels of Aβ in the brain are normally in the nanomolar range and up to 1 μM/kg even in APP-transgenic mice (Hsiao et al., 1996). Thus, only small concentrations of Aβ will likely bind to neprilysin under physiological and pathological conditions. Many issues should be solved to proceed to drug development of neprilysin activators.

Angiotensin-converting enzyme (ACE) is another Aβ-degrading agent. Captopril, a blood–brain barrier (BBB) penetrating ACE inhibitor, increases Aβ accumulation (Zou et al., 2007), and ACE overexpression in myelomonocytes reduces Aβ deposition in AD model mice (Bernstein et al., 2014). However, brain ACE deficient mice showed no significant alteration in endogenous Aβ levels (Eckman et al., 2006). In addition, two small studies assessing the clinical use of ACE inhibitors, found that they did not deteriorate dementia in AD and amnestic mild cognitive impairment (MCI) patients (Ohrui et al., 2004; Rozzini et al., 2006). Because of such conflicting findings, contributions of ACE to Aβ degradation in the brain *per se* remain ambiguous.

Insulin-degrading enzyme (IDE) is also known to have Aβ-degrading properties, and hyperinsulinemia in diabetes mellitus competitively inhibits Aβ degradation (Craft and Watson, 2004; Qiu and Folstein, 2006). Indeed, IDE deficient mice demonstrate increased cerebral accumulation of endogenous Aβ with hyperinsulinemia and glucose intolerance (Farris et al., 2003), and IDE overexpression ameliorates Aβ pathology (Leissring et al., 2003), suggesting a link between insulin metabolism and Aβ degradation. However, clinical evidence is still lacking and further studies on the association of IDE with AD pathogenesis may uncover potential treatment targets in AD. Some researchers have labeled AD “type 3 diabetes” (de la Monte and Wands, 2008). If hyperinsulinemia is related to resistance of neuronal cells to insulin, impaired insulin signaling in neurons is thought to lead to neuronal disturbances. A clinical trial assessing intranasal insulin therapy in the treatment of AD and amnestic MCI is anticipated to further elaborate on the relationship between AD and insulin signaling (Craft et al., 2012).

## Transcytotic Delivery

The cerebral vasculature originates from large arteries, such as middle cerebral artery and the circle of Willis. These arteries branch into the leptomeningeal arteries, which travel on the surface of the brain in the subarachnoid space. Leptomeningeal arteries further branch into smaller arteries and arterioles consisting of three layers: tunica intima (endothelium), tunica media (smooth muscle cells), and tunica adventitia (mainly collagen fibers). Finally, the terminals of arterioles become capillaries. Capillary lumen and brain parenchyma are separated by the BBB, which prevents the passive exchange of solutes between blood and brain (Iadecola, 2004).

Lipoprotein receptor-related protein-1 (LRP-1), a multifunctional scavenger and signaling receptor, is expressed in neural cells and cerebral microvessels including capillaries, small venules, and arterioles (Wolf et al., 1992; Tooyama et al., 1995; Shibata et al., 2000). LRP-1 has received increasing attention as it mediates transport of Aβ out of the brain across the BBB (Bell and Zlokovic, 2009). Many reports have described the genetic linkage of LRP-1 with AD (Kang et al., 1997; Lambert et al., 1998; Wavrant-DeVrièze et al., 1999) and CAA (Christoforidis et al., 2005). Colocalization of LRP-1 with Aβ was pathologically recognized in senile plaques (Rebeck et al., 1993; Donahue et al., 2006), strengthening the linkage. The relationship is further supported by reduced LRP-1 staining in vessels both in AD patients (Shibata et al., 2000; Donahue et al., 2006) and CAA model mice carrying a vasculotropic Dutch/Iowa mutant form of APP gene (Deane et al., 2004).

Animal experiments have confirmed the importance of transcytosis in the regulation of cerebral Aβ levels. Five hours after microinjection of ^125^I-labeled Aβ_1–40_ into the caudate nucleus, 73.8% of labeled tracer had been found in blood across the BBB in young wild-type mice, while ^125^I-labeled Aβ_1–40_ in cerebrospinal fluid (CSF) measured 10.7%, and only 15.6% of the dose remained in the brain parenchyma (Shibata et al., 2000). These findings suggest that endothelial transcytosis by LRP-1 and others is probably one of the most prominent pathways in cerebral Aβ clearance, although this study might underestimate other clearance pathways as all the Aβ peptides found in blood are considered to derive from transcytotic delivery.

LRP-1 binds to Aβ directly (Deane et al., 2004), but also binds indirectly via its ligands including α2-macroglobulin, receptor-associated protein, and apolipoprotein E (ApoE) (Narita et al., 1997; Bu, 2009; Kanekiyo and Bu, 2009). ApoE is the main chaperone of Aβ in central nervous system (Holtzman et al., 2012; Zolezzi et al., 2014). To date, three isoforms of ApoE have been described (ε2, ε3, and ε4), and the ApoE ε4 variant is considered to be one of the most relevant risk factors for AD and CAA (Premkumar et al., 1996; Zolezzi et al., 2014). ApoE immunoreactivity is common in amyloid plaques, suggesting that ApoE interacts with Aβ directly in AD brains and could strongly influence the rate of Aβ removal (Namba et al., 1991; Holtzman et al., 2012). Several authors have proposed ApoE as therapeutic target for Aβ clearance (Cramer et al., 2012; Zolezzi and Inestrosa, 2014). Cramer et al. reported that bexarotene, a retinoid X receptor agonist, stimulated the ApoE-dependent Aβ clearance through the actions of liver X receptors and peroxisome proliferator-activated nuclear receptor gamma in AD model mice (Cramer et al., 2012). As a result, cognitive deficits improved with reduced burden of Aβ plaque. However, some conflicting reports have been also documented (Fitz et al., 2013; Price et al., 2013; Tesseur et al., 2013; Veeraraghavalu et al., 2013). Further analysis and experimentation should be performed.

Receptor for advanced glycation end products (RAGE), an immunoglobulin supergene family member, is also known to be a key molecule in Aβ transcytosis (Yan et al., 2012). Strong staining for RAGE has been reported in the vessels of AD patients (Yan et al., 1996; Donahue et al., 2006) and has been shown to mediate influx of circulating Aβ into brain across the BBB (Deane et al., 2003). In addition, RAGE contributes to Aβ-related synaptic dysfunction and microglial activation (Yan et al., 1996; Origlia et al., 2008, 2010). These findings suggest that RAGE could be a therapeutic target in AD and CAA. Indeed, a RAGE inhibitor ameliorated cerebral Aβ burden and normalized cognitive performance in APP-transgenic mice (Deane et al., 2012). The phase III 18 month clinical trial of the RAGE inhibitor TTP488 is being planned for mild to moderate AD patients (The U.S. National Institutes of Health, 2014); positive results in phase II testing have been reported (Burstein et al., 2014).

## Perivascular Drainage

The central nervous system is devoid of conventional lymphatic vessels, unlike other organs containing networks of lymphatic vessels, which process various substances, such as wastes, fluid, proteins, and cells from tissues to lymph nodes. However, the lymphatic perivascular drainage system in the brain performs the main function assigned to systemic lymphatic vessels. Analysis of the lymphatic perivascular drainage system dates back as far as the nineteenth century, where it was shown that Indian ink injected into cisterna magna drained to the cervical lymph nodes (Schwalbe, 1869; Weller et al., 2010).

The detail of perivascular drainage system has been examined mainly by intracranial injection of various tracers, including ^125^I-labeled albumin (Bradbury et al., 1981; Szentistványi et al., 1984; Yamada et al., 1991), Indian ink (Zhang et al., 1992), and various fluorescent tracers (Carare et al., 2008). Recently, this drainage pathway was also confirmed by multi-photon imaging (Arbel-Ornath et al., 2013).

Fluorescent tracers, injected to the striatum, spread diffusely through the extracellular spaces of the brain parenchyma and enter the walls of blood vessels almost immediately. Confocal microscopy showed tracers colocalize with laminin in the basement membranes of capillary walls. Injected tracers were cleared from the basement membranes in the walls of capillaries and arteries, while some tracers were taken up by smooth muscle cells and perivascular macrophages (Zhang et al., 1992; Carare et al., 2008). Studies using radiolabeled tracers showed that drainage of interstitial fluid (ISF) and solutes continues along tunica media and the tunica adventitia of leptomeningeal and major cerebral arteries, through the base of the skull to the deep cervical lymph nodes (Szentistványi et al., 1984; Weller et al., 2010). Tissue soluble Aβ was detected by enzyme immunoassay in meningeal arteries and intracranial arteries but not in extracranial vessels (Shinkai et al., 1995). The clearance system leading to cervical lymph nodes was confirmed by subsequent injection into the inferior colliculus (Ball et al., 2010). Theoretical models have indicated that arterial pulsations could be the motive force behind ISF and solutes being driven centrifugally from the brain by reflection waves that follow each cardiac pulse wave (Schley et al., 2006).

This drainage route closely corresponds with the distribution of Aβ in the basement membranes of capillary and artery walls in CAA (Weller et al., 1998), which implies that the congestion of drainage pathway is associated with the pathogenesis of CAA. The fact that CAA was accelerated in the brains of immunized AD patients and that the CSF Aβ concentration was decreased both in AD and CAA patients may result from an impaired perivascular drainage pathway (Nicoll et al., 2004; Patton et al., 2006; Verbeek et al., 2009). Consistent with this, perivascular drainage of solutes is impaired in the aging mouse brain and in the presence of CAA (Hawkes et al., 2011). The fact that cerebral Aβ clearance was delayed after photothrombosis within individual vessels or middle cerebral artery occlusion (Garcia-Alloza et al., 2011), and after bilateral common carotid artery stenosis (Okamoto et al., 2012), further supports the notion that brain ischemia and impaired arterial pulsation could be an exacerbation factor of CAA. Consistent with the experimental data is the clinical finding that arterial stiffness, indicated by pulse wave velocity, has been associated with Aβ deposition in the brains of non-demented elderly adults (Hughes et al., 2013). Therefore, vasoactive drugs could have potential in the improvement of lymphatic congestion and facilitation of Aβ clearance in the brain.

## Convincing Effects of Phosphodiesterase Inhibitor

**Figure 3:**
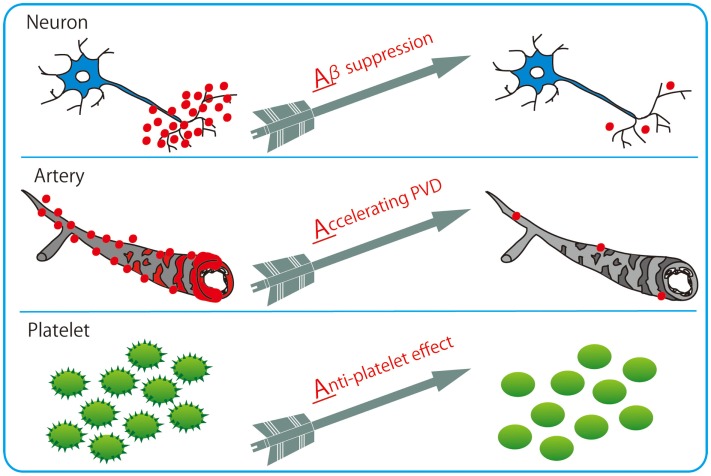
**Cilostazol with 3 Arrows: triple effects toward potential resolution of dementia**. Cilostazol, a selective inhibitor of PDE3, has pleiotropic capabilities of suppressing Aβ production in neurons, enhancing Aβ clearance through perivascular drainage system, and inhibiting platelet aggregation (anti-platelet effects).

**Figure 4:**
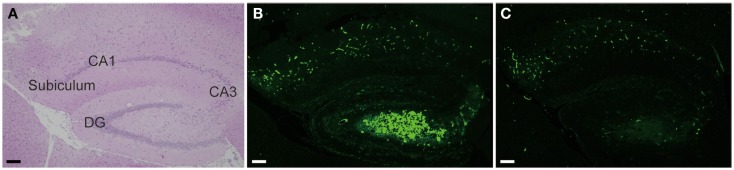
**Cilostazol reduced Aβ deposition**. Hippocampal images obtained from 17-month-old homozygous Tg-SwDI mice, a model of CAA, treated with vehicle **(A,B)** or cilostazol **(C)** for 15 months show that cilostazol treatment reduced levels of Aβ deposits in the hippocampus compared with vehicle treatment. Scale bars indicate 100 μm. **(A)** HE staining. **(B,C)** Thioflavin-S staining.

Among varieties of vasoactive drugs, cilostazol, a selective inhibitor of type 3 phosphodiesterase (PDE), is likely to be a promising agent for AD and CAA (Figure 3). PDE3 can hydrolyze both cAMP and cGMP, while increasing cAMP level is a major pharmacological effect of cilostazol (Ikeda, 1999). PDE3 is widely expressed in central nervous system and up-regulated in Aβ-positive vessels, especially in vascular smooth muscle cells (vSMC) (Maki et al., 2014), suggesting the possibility that PDE3 inhibition could be therapeutic for CAA. Cilostazol possesses multiple effects, such as increasing pulse rate (Shinohara et al., 2010) and arterial elasticity (Han et al., 2013), prolonging pulse duration time (Aruna and Naidu, 2007), and dilating cerebral vessels (Tanaka et al., 1989; Birk et al., 2004a,b); such vasoactive actions may promote efficiency of perivascular drainage. In support of this, clearance of fluorescent soluble Aβ tracers is significantly enhanced in cilostazol-treated CAA model mice, thereby resulting in maintenance of vascular integrity, amelioration of Aβ deposits (Figure 4), and prevention of cognitive decline (Maki et al., 2014). Memory-preserving activity of cilostazol has been demonstrated in aged wild-type mice (Yanai et al., 2014) and a rat model of chronic cerebral hypoperfusion (Watanabe et al., 2006), suggesting that cilostazol could be a potential disease modifying therapy of AD and other dementing disorders.

Recently, Nedergaad et al. suggested the “glymphatic pathway,” consisting of para-arterial CSF influx route, para-venous ISF efflux route, and convective bulk fluid flux (Iliff and Nedergaard, 2013; Nedergaard, 2013), as another clearance system in central nervous system. Aβ proteins may be cleared through this perivascular pathway, as well as the perivascular drainage system (Iliff et al., 2012), although the relationship to CAA pathogenesis remains to be clarified as Aβ does not accumulate in the venous system. Cerebral arterial pulsation with a vasoactive agent dobutamine drives perivascular CSF–ISF exchange (Iliff et al., 2013). Further investigation is required to determine whether other vasoactive drugs such as cilostazol could have a potential to facilitate paravascular clearance.

Many inhibitors of other PDE subtypes have been reported to produce cognitive enhancement (Reneerkens et al., 2009) and have been associated with neuronal cAMP signaling activation. Rolipram, a PDE4 inhibitor, reverses the decrease in cAMP regulatory element-binding protein (CREB) phosphorylation, which results in persistent improvement in synaptic function in AD model mice (Gong et al., 2004). Sildenafil, a PDE5 inhibitor, decreases Aβ levels in extracts of cerebral cortex and improves associative and spatial memory in AD model mice (Puzzo et al., 2009). Caffeine is a non-specific PDE inhibitor (Yoshimura, 2005), and its beneficial effects have been clarified in many clinical AD studies (Eskelinen et al., 2009; Eskelinen and Kivipelto, 2010). Caffeine stimulates cAMP-dependent protein kinase A signaling and increases CREB phosphorylation in AD model mice (Arendash et al., 2006; Zeitlin et al., 2011). Protein kinase A activation then suppresses the expression of Aβ-synthesizing enzymes such as β- and γ-secretase, leading to reduced Aβ production (Arendash et al., 2009). Cilostazol also reduces Aβ production *in vitro* (Lee et al., 2012, 2014; Maki et al., 2014), and suppresses Aβ-induced tauopathy and tau phosphorylation *in vitro* (Lee et al., 2012, 2014). However, as only a minor fraction of cilostazol passes through BBB (Akiyama et al., 1985), it remains to be elucidated whether these positive effects of cilostazol do occur in AD patients.

Cilostazol has a wide variety of pleiotropic effects capable of inducing neurogenesis (Lee et al., 2009; Tanaka et al., 2010), promoting oligodendrocyte precursor cell differentiation (Miyamoto et al., 2013), preventing lipid peroxidation (Hiramatsu et al., 2010; Kurtoglu et al., 2014), enhancing cholesterol efflux from macrophages (Nakaya et al., 2010), ameliorating insulin resistance (Wada et al., 2013), reducing inflammatory burden (Otsuki et al., 2001; Tsai et al., 2008; Hattori et al., 2009), and improving systemic lymphatic function by inducing proliferation and stabilization of lymphatic endothelial cells (Kimura et al., 2014). In a clinical setting, cilostazol is currently used as an anti-platelet drug (Gotoh et al., 2000; Shinohara et al., 2010), and may be used to prevent ischemic events in patients with CAA. Major manifestations of CAA include lobar hemorrhage and cortical microhemorrhage, as well as CMI. As most CAA patients are elderly (Zhang-Nunes et al., 2006), this necessitates the use of anti-platelet drugs with little risk of hemorrhage (Charidimou et al., 2012). The second Cilostazol Stroke Prevention Study (CSPS2) for patients with cerebral infarction showed that the hemorrhagic stroke was significantly less frequent in cilostazol treatment than with aspirin (Shinohara et al., 2010; Uchiyama et al., 2014). The prevention of cerebral hemorrhage may be explained by reproducible experimental evidence showing that cilostazol inhibits expression of matrix metalloproteinase-9 and protects vascular endothelial cells (Ishiguro et al., 2010; Hase et al., 2012; Kasahara et al., 2012). Endothelial protection with cilostazol mediates increase in nitric oxide, which dilates blood vessels (Oyama et al., 2011), leading to increased cerebral blood flow (Mochizuki et al., 2001; Matsumoto et al., 2011; Sakurai et al., 2013). These results suggest that cilostazol could be suitable for patients with both AD and CVD, the most common type of dementia in the elderly.

Favorable effects have already been described in observational clinical studies, which demonstrated the efficacy of cilostazol in patients with MCI (Taguchi et al., 2013), donepezil-treated patients with clinically probable AD (Arai and Takahashi, 2009; Ihara et al., 2014), and AD with CVD (Sakurai et al., 2013). Randomized placebo-controlled clinical trials are being planned for patients with MCI.

## Future Strategy for AD and CAA Treatment

Aging inevitably increases the amount of Aβ burden in the brain, implying a strong relationship between impaired Aβ metabolism and age (Funato et al., 1998). Since heterogeneity and multimorbidity are common in the elderly (Barnett et al., 2012), dementia likely originates from a combination of different pathological substrates. As the population ages, the distribution of AD shifts to older ages in developed countries (Hebert et al., 2013), resulting in an increasing number of demented patients with numerous complicated etiologies. Given that the balance between Aβ synthesis and clearance determines brain Aβ accumulation, and that Aβ is cleared by several pathways stated above, multi-drugs combination therapy would likely be necessary for sporadic AD with complicated etiologies. Combination therapy has already been applied to various diseases, such as hypertension, diabetes mellitus, and malignant tumors. The ultimate goal will be to develop a sovereign remedy of AD, and we hope that the recent rapid advances in drug development will enable us to delay the onset or modify the progression of cognitive impairment with multi-targeting therapies. Further investigation from various viewpoints will thus be essential for the development of novel treatment for AD and CAA.

## Conflict of Interest Statement

The authors declare that the research was conducted in the absence of any commercial or financial relationships that could be construed as a potential conflict of interest.
